# Serum Fibrinogen as a Biomarker for Disease Severity and Exacerbation in Patients with Non-Cystic Fibrosis Bronchiectasis

**DOI:** 10.3390/jcm11143948

**Published:** 2022-07-07

**Authors:** Seung Jun Lee, Jong Hwan Jeong, Manbong Heo, Sunmi Ju, Jung-Wan Yoo, Yi Yeong Jeong, Jong Deog Lee

**Affiliations:** Division of Pulmonology and Allergy, Department of Internal Medicine, Gyeongsang National University Hospital, Gyeongsang National University School of Medicine, Jinju 52727, Korea; juny2278@naver.com (S.J.L.); bechem6939@naver.com (J.H.J.); mabory@nate.com (M.H.); smangel518@naver.com (S.J.); chareok-sa@hanmail.net (J.-W.Y.); dr202202@naver.com (Y.Y.J.)

**Keywords:** fibrinogen, non-cystic fibrosis bronchiectasis, FACED score, bronchiectasis severity index

## Abstract

**Background**: Serum biomarkers associated with severe non-cystic fibrosis (CF) bronchiectasis are currently lacking. We assessed the association of serum fibrinogen, adiponectin, and angiopoietin-2 levels with the severity and exacerbation of bronchiectasis. **Methods**: Serum levels of fibrinogen, adiponectin, and angiopoietin-2 were measured and compared in patients with stable non-CF bronchiectasis (*n* = 61) and healthy controls (*n* = 16). The correlations between the three biomarkers and the bronchiectasis severity index (BSI) or FACED scores were assessed. Univariate and multivariate linear regression analyses were performed to identify variables independently associated with BSI and FACED scores in patients with bronchiectasis. Additionally, the exacerbation-free survival was compared between groups of patients with high and low fibrinogen levels, while the predictors of exacerbation were analyzed using Cox proportional hazards regression. **Results**: Patients with non-CF bronchiectasis carried higher fibrinogen (3.00 ± 2.31 vs. 1.52 ± 0.74 µg/mL; *p* = 0.016) and adiponectin (12.3 ± 5.07 vs. 9.17 ± 5.30 µg/mL; *p* = 0.031) levels compared with healthy controls. The serum level of angiopoietin-2 was comparable between the two groups (1.49 ± 0.96 vs. 1.21 ± 0.79 ng/mL, *p* = 0.277). Correlations of adiponectin and angiopoietin-2 with BSI and FACED scores were not significant. However, there were significant correlations between fibrinogen and both BSI (r = 0.428) and FACED scores (r = 0.484). Multivariate linear regression analysis revealed that fibrinogen level was an independent variable associated with both BSI and FACED scores. A total of 31 (50.8%) out of 61 patients experienced exacerbation during the follow-up period of 25.4 months. Exacerbation-free survival was significantly longer in patients with low fibrinogen levels than in those with high fibrinogen (log-rank test, *p* = 0.034). High fibrinogen levels and *Pseudomonas* colonization were independent risk factors for future exacerbation (HR 2.308; *p* = 0.03 and HR 2.555; *p* = 0.02, respectively). **Conclusions**: Serum fibrinogen, but not adiponectin or angiopoietin-2, is a potential biomarker closely associated with the severity and exacerbation of non-CF bronchiectasis.

## 1. Introduction

The interest and progress in the field of non-cystic fibrosis bronchiectasis (referred to as bronchiectasis), which was once regarded as an orphan disease for decades, are currently growing. Determination of disease severity is essential to monitor the disease activity and to predict the prognosis. The FACED instrument and bronchiectasis severity index (BSI) have been developed to assess the severity of bronchiectasis [1,2]. The FACED score and BSI are composed of five and nine variables, respectively. Although both scoring systems are excellent prognostic tools in bronchiectasis, it is not easy to calculate these scores every time in daily real practices. Furthermore, some patients diagnosed with bronchiectasis may not be able to expectorate sputum, which is essential to calculate the FACED score and BSI. To overcome these limitations, various attempts have been made to develop simple blood biomarkers associated with disease severity and prognosis of patients diagnosed with bronchiectasis. Saleh et al. measured blood concentrations of 31 candidate proteins and identified systemic inflammatory markers associated with the disease severity and etiology of bronchiectasis. They showed a heterogeneous systemic inflammatory response, with interleukin (IL)-17 distinguishing primary immunodeficiency from other etiologies and plasma fibrinogen associated with the severity of bronchiectasis [3]. Another important study by Huang et al. reported that serum desmosine levels in patients with stable bronchiectasis were associated with all-cause and cardiovascular mortality [4]. Our team also tested some blood biomarkers and demonstrated that serum albumin and serum hepatocyte growth factor are associated with severity and exacerbation in a cohort of patients with bronchiectasis at a single tertiary hospital in South Korea [5,6,7].

As a component of the authors’ biomarker study in bronchiectasis, serum levels of fibrinogen, adiponectin, and angiopoietin-2 were tested to determine their role as significant biomarkers. Fibrinogen is an acute-phase protein secreted in response to systemic inflammation as well as a major component of the coagulation cascade [5]. Numerous clinical studies analyzed blood fibrinogen in patients with chronic obstructive pulmonary disease (COPD), which is a chronic inflammatory airway disease similar to bronchiectasis [8,9]. Adiponectin is a protein hormone which acts as a major cytokine in metabolic syndrome [10]. Previous studies demonstrated that adiponectin was associated with the severity, lung function decline, and development of emphysema in patients with COPD [11]. Angiopoietin is a key protein in angiogenesis along with vascular endothelial growth factor. The angiogenic activity, which can be measured based on angiopoietin, was highlighted as an alternative pathophysiology of chronic lung disease such as COPD [12].

This study aimed to identify useful biomarkers that can be easily measured and are closely associated with the severity and exacerbation of bronchiectasis. We evaluated the potential of fibrinogen, adiponectin, and angiopoietin-2 as meaningful biomarkers of bronchiectasis in this study.

## 2. Methods

### Study Population

A total of 61 patients with bronchiectasis who visited the outpatient clinic at Gyeongsang National University Hospital (Jinju, Korea) from July 2016 to June 2018 were prospectively enrolled. Based on age, gender, and smoking history, 16 matched healthy subjects without underlying disorders who visited the health promotion center comprised the control group. We only included patients with stable bronchiectasis, which was confirmed by at least 4 weeks of no antibiotic or corticosteroid use. Bronchiectasis was diagnosed morphologically via high-resolution computed tomography of patients with compatible clinical history who met the diagnostic criteria [13]. The inclusion criteria based on high-resolution computed tomography were: (1) a larger internal diameter of bronchi than that of the accompanying pulmonary artery and (2) a lack of bronchial tapering in the lung periphery. The exclusion criteria were: (1) traction bronchiectasis due to interstitial lung disease and other pulmonary diseases; (2) patients with non-tuberculous mycobacterial lung disease, active pulmonary tuberculosis, or coexisting active malignant disease; (3) an inability to perform the pulmonary function test; and (4) an inability to expectorate sputum. The institutional review board of Gyeongsang National University Hospital approved this study (approval number IRB-2016-04-009). Written informed consent was obtained before peripheral blood sampling.

## 3. Study Design

Clinical information including age, gender, body mass index, smoking status, and comorbid diseases was recorded on the day of study recruitment. Laboratory values preferentially selected as biomarkers of systemic inflammation included white blood cells (WBCs) with a differential cell count, platelets, albumin, uric acid, bilirubin, and C-reactive protein (CRP) along with new candidate biomarkers including fibrinogen, adiponectin, and angiopoietin-2. Additional clinical variables were also acquired to calculate BSI and FACED scores, including *Pseudomonas* colonization, colonization with other organisms, the Medical Research Council (MRC) dyspnea scale, forced expiratory volume in 1 s (FEV_1_), the number of affected lobes, hospital admission, and disease exacerbation. Peripheral blood sampling was performed when the patients’ condition was clinically stable during their regular outpatient visits and when control subjects visited the health promotion center. Patients’ comorbidities included COPD, bronchial asthma, hypertension, cardiovascular disease, diabetes, and a previous history of tuberculosis. The diagnosis of COPD was established via spirometry if the post-bronchodilator FEV_1_/forced vital capacity (FVC) ratio was <0.7 according to the international guidelines. Exacerbation of bronchiectasis was defined according to the consensus definition criteria established by experts in bronchiectasis [14]. The radiological severity of bronchiectasis was determined using a modified Reiff score, which was based on the number of affected lobes and pattern of bronchiectasis [13]. Spontaneous sputum samples were used to assess the sputum bacteriology. The isolation of bacteria in sputum culture more than twice at intervals of more than 3 months indicated chronic colonization. The BSI and FACED scores were calculated based on patients’ clinical variables and severity classified into three groups: low (BSI, 0–4; FACED score, 0–2), intermediate (BSI, 5–8; FACED score, 3–4), and high (BSI, 9 or more; FACED score, 5–7) based on the original studies [1,2]. All the patients were monitored regularly at 3–4-month intervals and exacerbations were recorded.

## 4. Measurement of Serum Fibrinogen, Adiponectin, and Angiopoietn-2 Levels

Approximately 10 mL of peripheral venous blood was sampled and serum was collected by centrifugation for 15 min at 1000× *g* at room temperature to measure fibrinogen, adiponectin, and angiopoietin-2 levels. The serum was aliquoted and stored at −80 °C until use. The levels of fibrinogen, adiponectin, and angiopoietin-2 were measured using an enzyme-linked immunosorbent assay kit (Human fibrinogen SimpleStep ELISA kit, Human adiponectin SimpleStep ELISA kit, and Angiopoietein 2 Human ELISA kit, Abcam, Boston, MA, USA) according to the manufacturer’s instructions.

## 5. Statistical Analysis

Data are presented as the mean ± standard deviation or as frequency and percentage. An unpaired *t*-test was used to compare continuous variables of bronchiectasis patients with healthy controls. The correlation coefficients and significance of continuous variables were analyzed using the Pearson correlation test. The continuous variables of the three severity groups were compared using one-way analysis of variance with a Bonferroni *post hoc* test. Multiple linear regression analysis was performed to identify variables independently associated with the dependent variables including the BSI and FACED scores. Only statistically significant variables in the univariate analysis were included in the multivariate analysis. Patients with bronchiectasis were dichotomized into two groups according to fibrinogen levels to evaluate the effectiveness of serum fibrinogen as a predictive factor of exacerbation. The median level (2.182 µg/mL) of fibrinogen in enrolled patients with bronchiectasis and control subjects was subjectively chosen by authors and used as a cut-off value between high- and low-fibrinogen groups. Predictive factors independently associated with future exacerbation were analyzed via univariate Cox proportional hazards regression and stepwise multivariate analysis. Hazard ratios (HRs) and 95% confidence intervals (CIs) were calculated. The probability of exacerbation between groups of patients with high and low fibrinogen was calculated using Kaplan–Meier method. The differences were assessed using the log-rank test. The follow-up time for analysis of exacerbation was defined as the time from the date of blood sampling to the date of first exacerbation, or the date of the last outpatient visit. Statistical significance was accepted at *p* < 0.05. Statistical analyses were performed using SPSS 21.0 for Windows (SPSS, Chicago, IL, USA).

## 6. Results

### 6.1. Baseline Demographic and Clinical Characteristics

The study population comprised 61 patients diagnosed with bronchiectasis and 16 control subjects. The mean age of patients with bronchiectasis was 65.3 ± 9.12 years, and 19 patients (31.1%) were male. The mean body mass index was 22.6 ± 3.44 kg/m^2^ and the mean percent predicted value of FEV_1_ was 61.8 ± 20.1%. Forty-nine patients diagnosed with bronchiectasis (80.3%) were never smokers. Clinical variables of patients with bronchiectasis used to calculate BSI and FACED scores are shown in Table 1. The mean BSI and FACED scores were 8.00 ± 3.96 and 2.10 ± 1.83, respectively. COPD was the most common comorbid disease, followed by bronchial asthma, hypertension, a previous history of tuberculosis, diabetes, and cardiovascular disease. The mean age of the matched control subjects was 63.6 ± 3.42 years and 6 (37.5%) out of 16 subjects were male. Body mass index and smoking status were comparable between patients with bronchiectasis and control subjects.

### 6.2. Comparison of Biomarkers between Patients with Bronchiectasis and Control Subjects, Correlation between Biomarkers and Severity Scores, and Differences in Biomarker Levels Associated with Severity

**Figure 1:**
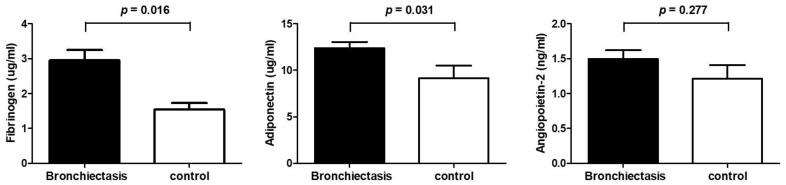
Comparison of fibrinogen, adiponectin, and angiopoietin-2 levels between patients with bronchiectasis and healthy controls.

**Figure 2:**
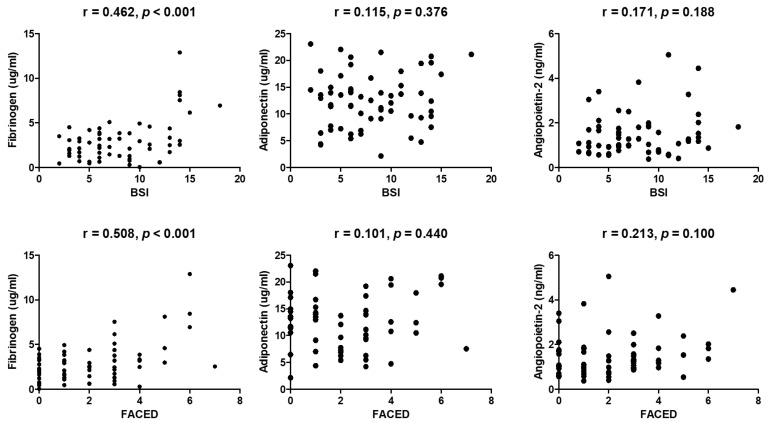
Correlations of fibrinogen, adiponectin, and angiopoietin-2 with BSI and FACED score. BSI: bronchiectasis severity index; FACED: FACED score.

**Figure 3:**
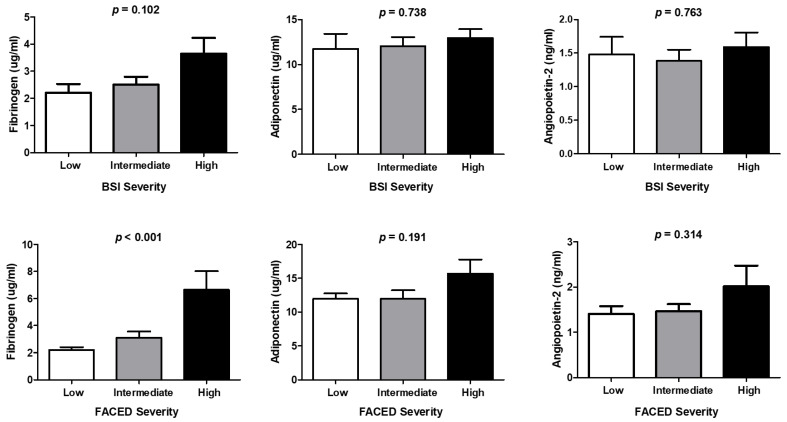
Levels of fibrinogen, adiponectin, and angiopoietin-2 across severity groups of BSI and FACED score. BSI: bronchiectasis severity index; FACED: FACED score.

Patients with bronchiectasis had significantly higher plasma fibrinogen (2.95 ± 2.30 vs. 1.54 ± 0.74 µg/mL, *p* = 0.016) and adiponectin (12.3 ± 5.10 vs. 9.17 ± 5.30 µg/mL, *p* = 0.031) levels compared with control subjects. The plasma levels of angiopoietin-2 did not differ significantly between the two groups (1.49 ± 0.96 vs. 1.21 ± 0.79 ng/mL, *p* = 0.277) (Figure 1). Correlations of adiponectin and angiopoietin-2 levels with BSI or FACED score in patients with bronchiectasis were not significant. However, significant correlation existed between fibrinogen and both BSI (r = 0.462, *p* < 0.001) and FACED score (r = 0.508, *p* < 0.001) (Figure 2). The fibrinogen level varied significantly based on mild, intermediate, and severe FACED score (*p* < 0.001). However, no significant differences in the BSI score of the fibrinogen level were found across severity groups. Differences in BSI or FACED score based on adiponectin and angiopoietin levels were not significant across severity groups (Figure 3).

### 6.3. Independent Factors Associated with Severity Scoring Systems

The univariate linear regression analysis revealed that the serum fibrinogen level was significantly associated with both BSI and FACED scores. Among various laboratory variables, serum albumin and CRP levels were significantly associated with BSI score. Factors significantly associated with FACED score were WBC count, platelet-to-lymphocyte ratio, uric acid level, and CRP level. Following multivariate linear regression analysis, fibrinogen level was the only independent variable associated with BSI score (β coefficient = 0.646, *p* = 0.002). Additionally, fibrinogen level and WBC count were independently associated with FACED score (β coefficient = 0.348, *p* < 0.001 and β coefficient = 0.261, *p* = 0.01, respectively) (Table 2).

### 6.4. Exacerbation

**Figure 4:**
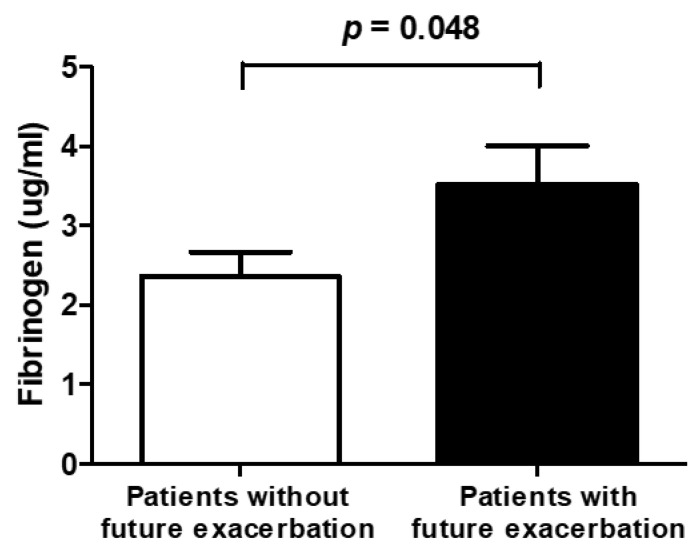
Comparison of fibrinogen levels between patients with and without exacerbation.

**Figure 5:**
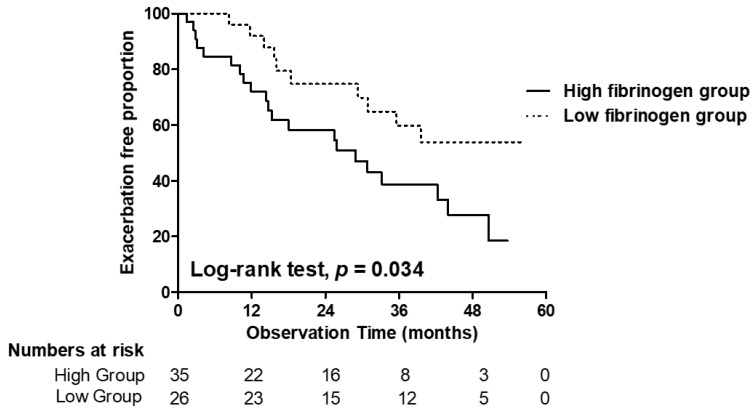
Kaplan–Meier curves for exacerbation-free survival between groups of patients with low and high fibrinogen.

The median follow-up interval from biomarker sampling to exacerbation or the last out-patient visit was 25.4 months. A total of 31 (50.8%) out of 61 patients experienced one or more exacerbations during the follow-up period. The baseline fibrinogen level was significantly higher in patients with future exacerbation compared with those without (3.52 ± 2.68 vs. 2.36 ± 1.69 µg/mL, *p* = 0.048) (Figure 4). Kaplan–Meier survival analysis revealed that the exacerbation-free survival was significantly longer in patients with low fibrinogen levels compared with those carrying high fibrinogen levels (log-rank test, *p* = 0.034) (Figure 5). The univariate Cox proportional hazards model for exacerbation during the follow-up period showed that high fibrinogen levels and chronic colonization with the *Pseudomonas* organism were significantly associated with future exacerbation in patients with bronchiectasis. High levels of adiponectin and angiopoietin-2 were not associated with exacerbation. High fibrinogen level and *Pseudomonas* colonization were statistically significant in the multivariate analysis and were thus independent predictive factors of future exacerbation: high fibrinogen level (HR, 2.555; *p* = 0.02) and *Pseudomonas* colonization (HR, 2.308; *p* = 0.03) (Table 3).

## 7. Discussion

This study investigated the clinical significance of serum fibrinogen, adiponectin, and angiopoietin-2 as biomarkers of bronchiectasis. The following evidence in this study supports the usefulness of fibrinogen: (i) higher serum levels of fibrinogen in stable bronchiectasis than in healthy controls; (ii) independent correlation between fibrinogen and disease severity scores; and (iii) significant association between high fibrinogen levels and future exacerbation. However, the study does not reveal the clinical significance of adiponectin and angiopoietin-2.

Fibrinogen is a key cogulation factor. However, it also acts as an acute-phase reactant protein. Blood fibrinogen was evaluated as a prognostic biomarker of several solid malignancies. A meta-analysis showed that elevated levels of fibrinogen were poor prognostic factors of overall survival in patients with cancers of the lung and liver [15,16]. Blood fibrinogen is a useful biomarker in COPD and is thus utilized as a drug development tool [17]. According to a recent meta-analysis, high fibrinogen level was associated with exacerbation and mortality in patients with COPD [18]. Fibrinogen may represent a potential biomarker in bronchiectasis considering that bronchiectasis and COPD are chronic airway diseases caused by neutrophil-dominant airway inflammation with concomitant systemic inflammation. Saleh et al. previously evaluated blood levels of several candidate proteins in bronchiectasis and found that fibrinogen was the most promising protein associated with disease severity [3]. Results of the present study reinforced the association between blood fibrinogen levels and the severity of bronchiectasis. Furthermore, this study validated fibrinogen as an independent factor associated with disease severity after adjusting for other laboratory variables, and serum fibrinogen was associated with futere exacerbations of bronchiectasis.

We evaluated several markers of systemic inflammation such as WBCs with a differential cell count, platelets, albumin, uric acid, bilirubin, and CRP in this study. Coban et al. reported that CRP was significantly associated with BSI and FACED score, and Aliberti et al. reported that elevated platelet count was associated with disease severity, exacerbation, and mortality in stable bronchiectasis [19,20]. In this study, total WBC count was independently associated with FACED score, whereas other laboratory variables were not associated with disease severity in bronchiectasis.

Sputum neutrophil elastase activity is a representative biomaker of bronchiectasis. Neutrophil elastase is one of the neutrophil serine proteases involved in neutrophil degradation during the inflammatory response. A prospective study by Chalmers et al. showed that neutrophil elastase activity in sputum was associated with the BSI, MRC dyspnea score, FEV_1_, and radiologic severity in bronchiectasis among patients in Scotland. Futhermore, sputum elastase activity showed good discrimination between severe exacerbation and overall survival [21]. Shortly thereafter, an external validation of the Scottish study was conducted in Southern Europe, including Italy and Spain. Levels of sputum neutrophil elastase were significantly correlated with the severity of bronchiectasis, chronic *Pseudomonas* infection, and quality of life in patients with bronchiectasis [22]. Based on the critical role of neutrophil elastase in bronchiectasis, bresocatib, a selective and reversible inhibitor of dipeptidyl peptidase 1 that inhibits neutrophil elastase activity, was developed as an effective new drug for the treatment of bronchiectasis [23]. The present study demonstrates serum fibrinogen as an excellent biomarker of bronchiectasis, similar to sputum neutrophil elastase, although this study failed to evaluate decline in lung function or long-term mortality.

One of the interesting findings in this study is that COPD was the most common comorbid disease in patients diagnosed with bronchiectasis. COPD coexisted with bronchiectasis in more than 50% of the enrolled patients, although the majority (80.3%) of patients were never smokers. The risk factor of developing COPD in these patients might be a limitation of lung growth and development due to recurrent respiratory infections during childhood. Despite the unclear causal relationship between bronchiectasis and COPD, bronchiectasis is considered as a risk factor for COPD in never smokers.

Some limitations of this study need to be acknowledged. First, this study enrolled a small number of patients with bronchiectasis and control subj0ects because this study was conducted in a single university-affiliated hospital in South Korea. A multi-center study including a large number of patients and controls is therefore needed. Second, no external validation of fibrinogen effectiveness was performed. Third, the cut-off of serum fibrinogen between high- and low-fibrinogen groups was somewhat arbitrary. The median level of patients with bronchiectasis and control subjects was used as a cut-off in this study; however, we cannot assure the accuracy of the cut-off value. Despite these limitations, this is the first prospective observational study showing that serum fibrinogen in patients with bronchiectasis is not only independently associated with disease severity scores indirectly, but is also a direct predictor of exacerbation.

In conclusion, serum fibrinogen level, but not adiponectin or angiopoietin-2, may represent a potential biomarker of disease severity and exacerbation of bronchiectasis.

## Figures and Tables

**Table 1:** Baseline characteristics of study patients.

Characteristics	Total Patients (*n* = 61)
Age, years	65.3 ± 9.12
Male	19 (31.1)
Body mass index, kg/m^2^	22.6 ± 3.44
FEV_1_/FVC, %	65.3 ± 12.9
FEV_1,_ L	1.54 ± 0.62
FEV_1_, %	61.8 ± 20.2
Smoking status Current smoker Former smoker Never smoker	3 (4.9) 9 (14.8) 49 (80.3)
History of respiratory hospitalization Yes No	23 (37.7) 38 (62.3)
History of acute exacerbation Yes No	36 (59.0) 25 (41.0)
Number of exacerbations in previous year	0.97 ± 1.21
Chronic colonization with Pseudomonas Yes No	12 (19.7) 49 (80.3)
Number of affected lobes	2.90 ± 1.34
Pattern of bronchiectasis Cylindrical Varicose Cystic	24 (39.3) 4 (6.6) 33 (54.1)
FACED score	2.10 ± 1.83
BSI	8.00 ± 3.96
Comorbid diseases COPD Bronchial asthma Hypertension Previous history of tuberculosis Diabetes Cardiovascular disease	32 (52.5) 15 (24.6) 14 (23.0) 9 (14.8) 5 (8.2) 3 (4.9)

Data are presented as mean ± SD or number (%). FEV_1_, forced expiratory volume in 1 s; FVC, forced vital capacity; BSI, bronchiectasis severity index; COPD, chronic obstructive pulmonary disease.

**Table 2:** Univariate and multivariate linear regression analysis of laboratory variables associated with BSI and FACED score.

Dependent Variables	Explanatory Variables	Univariate Analysis			Multivariate Analysis		
		β Coefficient	95% CI	*p*-Value	β Coefficient	95% CI	*p*-Value
BSI	Fibrinogen, µg/mL	0.794	0.397; 1.191	<0.001	0.646	0.244; 1.149	0.002
	Albumin, g/dL	−2.909	−5.537; −0.280	0.031	−1.512	−3.965; 0.940	0.222
	CRP, mg/L	0.189	0.057; 0.321	0.006	0.118	−0.010; 0.246	0.071
FACED score	Fibrinogen, µg/mL	0.404	0.226; 0.583	<0.001	0.348	0.171; 0.525	<0.001
	WBC, 10^9^/L	0.295	0.074; 0.516	0.01	0.261	0.066; 0.456	0.01
	PLR	0.014	0.002; 0.025	0.022	0.003	−0.008; 0.015	0.573
	Uric acid, mg/dL	0.424	0.089; 0.759	0.014	0.230	−0.063; 0.523	0.121
	CRP, mg/L	0.080	0.018; 0.142	0.012	0.026	−0.034; 0.087	0.385

BSI, bronchiectasis severity index; CI, confidence interval; CRP, c-reactive protein; WBC, white blood cell; PLR, platelet-to-lymphocyte ratio.

**Table 3:** Univariate and multivariate Cox proportional hazards model of exacerbation during the observation period.

Variables	Univariate Analysis			Multivariate Analysis		
	HR	95% CI	*p*-Value	HR	95% CI	*p*-Value
Age, years	1.015	0.978–1.053	0.424			
Sex						
Male	Ref	1				
Female	1.086	0.508–2.323	0.831			
Body mass index, kg/m^2^	0.970	0.873–1.077	0.564			
FEV_1_% predicted	0.991	0.971–1.012	0.406			
Hospital admission before study						
No	Ref	1				
Yes	1.656	0.814–3.372	0.164			
Exacerbations before the study						
0–2	Ref	1				
3 or more	1.197	0.490–2.925	0.694			
mMRC dyspnea score						
0–1	Ref	1				
≥2	0.871	0.332–2.183	0.779			
Pseudomonas colonization						
No	Ref	1			1	
Yes	2.420	1.103–5.308	0.027	2.555	1.116–5.620	0.02
Number of affected lobes						
0–2 lobes	Ref	1				
≥3 lobes	1.245	0.555–2.790	0.595			
Fibrinogen						
Low (<2.182 µg/mL)	Ref	1			1	
High (≥2.182 µg/mL)	2.215	1.041–4.713	0.039	2.308	1.083–4.919	0.03
Adiponectin						
Low (<11.447 µg/mL)	Ref	1				
High (≥11.447 µg/mL)	0.986	0.476–2.039	0.969			
Angiopoietin-2						
Low (<1.177 ng/mL)	Ref	1				
High (≥1.177 ng/mL)	1.117	0.550–2.269	0.759			

FEV_1_: forced expiratory volume in 1 s; mMRC: modified Medical Research Council; HGF: hepatocyte growth factor; HR: hazard ratio; CI: confidence interval.

## Data Availability

The datasets analyzed during the current study are available from the Internal Medicine of Gyeongsang National University Hospital on reasonable request.
